# The First Insensibility from Ether

**Published:** 1879-12

**Authors:** 


### THE FIRST INSENSIBILITY FROM ETHER.

FOR the short operations of minor surgery, and the reductions of dislocations, or opening of abscesses, it is extremely useful and of every-day application. Such a patient wishes to be operated upon without pain, or from being incapacitated from attending to business during the remainder of the day. He lies down upon the sofa, and with one hand places the ether inhaler, on a sponge wet with ether, over his face, mouth and nose, and holds the other arm and hand up in the air.

This arm, after the ether has been breathed for a few minutes, will drop, and from thirty to fifty seconds of unconsciousness will be had, in which to operate. The sponge being removed, the patient is ready to go about his business. It gives rise to no headache, nausea, or other unpleasant symptoms, and is particularly useful in children. The chief source of disappointment is in not recognizing the right moment, for if this is allowed to pass, unconsciousness will not occur until full etherization. The first insensibility is sure to come. When the arm moves, be ready, and as soon as it drops perform the operation; no pain will be felt.—*Medical Times.*

